# A Multiscale Agent-Based *in silico* Model of Liver Fibrosis Progression

**DOI:** 10.3389/fbioe.2014.00018

**Published:** 2014-05-30

**Authors:** Joyeeta Dutta-Moscato, Alexey Solovyev, Qi Mi, Taichiro Nishikawa, Alejandro Soto-Gutierrez, Ira J. Fox, Yoram Vodovotz

**Affiliations:** ^1^Department of Biomedical Informatics, University of Pittsburgh, Pittsburgh, PA, USA; ^2^Department of Surgery, University of Pittsburgh, Pittsburgh, PA, USA; ^3^Center for Inflammation and Regenerative Modeling, McGowan Institute for Regenerative Medicine, University of Pittsburgh, Pittsburgh, PA, USA; ^4^Department of Mathematics, University of Pittsburgh, Pittsburgh, PA, USA; ^5^Department of Sports Medicine and Nutrition, University of Pittsburgh, Pittsburgh, PA, USA; ^6^McGowan Institute for Regenerative Medicine, University of Pittsburgh, Pittsburgh, PA, USA; ^7^Department of Surgery, Children’s Hospital of Pittsburgh, Pittsburgh, PA, USA; ^8^Department of Pathology, University of Pittsburgh, Pittsburgh, PA, USA; ^9^Thomas E. Starzl Transplantation Institute, University of Pittsburgh, Pittsburgh, PA, USA

**Keywords:** cirrhosis, computer simulation, inflammation, elastography, hepatocyte

## Abstract

Chronic hepatic inflammation involves a complex interplay of inflammatory and mechanical influences, ultimately manifesting in a characteristic histopathology of liver fibrosis. We created an agent-based model (ABM) of liver tissue in order to computationally examine the consequence of liver inflammation. Our liver fibrosis ABM (LFABM) is comprised of literature-derived rules describing molecular and histopathological aspects of inflammation and fibrosis in a section of chemically injured liver. Hepatocytes are modeled as agents within hexagonal lobules. Injury triggers an inflammatory reaction, which leads to activation of local Kupffer cells and recruitment of monocytes from circulation. Portal fibroblasts and hepatic stellate cells are activated locally by the products of inflammation. The various agents in the simulation are regulated by above-threshold concentrations of pro- and anti-inflammatory cytokines and damage-associated molecular pattern molecules. The simulation progresses from chronic inflammation to collagen deposition, exhibiting periportal fibrosis followed by bridging fibrosis, and culminating in disruption of the regular lobular structure. The ABM exhibited key histopathological features observed in liver sections from rats treated with carbon tetrachloride (CCl_4_). An *in silico* “tension test” for the hepatic lobules predicted an overall increase in tissue stiffness, in line with clinical elastography literature and published studies in CCl_4_-treated rats. Therapy simulations suggested differential anti-fibrotic effects of neutralizing tumor necrosis factor alpha vs. enhancing M2 Kupffer cells. We conclude that a computational model of liver inflammation on a structural skeleton of physical forces can recapitulate key histopathological and macroscopic properties of CCl_4_-injured liver. This multiscale approach linking molecular and chemomechanical stimuli enables a model that could be used to gain translationally relevant insights into liver fibrosis.

## Introduction

Fibrosis is an aberrant wound-healing response characterized by excessive deposition of scar tissue composed of extracellular matrix (ECM) proteins. In the liver, fibrosis is caused by chronic inflammation arising from viral hepatitis, alcohol, drugs, and metabolic or autoimmune diseases (Friedman, 2008). Progressive fibrosis distorts liver vasculature and architecture, leading to cirrhosis (Schuppan and Afdhal, 2008). Secondary effects of liver cirrhosis result in approximately 35,000 deaths each year in the US (1.2% of all US deaths). As organ transplant is the only available treatment for cirrhosis, a better understanding of fibrosis is needed. Decades of research have elucidated many cellular effectors and key cytokines regulating the fibrotic process, the interplay of inflammation and repair, and determinants of ECM turnover (Iredale, 2007). However, translation of this knowledge into anti-fibrotic therapies remains a challenge, as fibrotic pathology in humans can only be observed in diagnostic biopsies, which are invasive, risky procedures, and are usually performed on patients in advanced stages of disease (Popov and Schuppan, 2009).

Inflammation and fibrosis are closely linked. Injury elicits recruitment of inflammatory cells to the liver, as well as activation of resident inflammatory cells, and a close topographical relationship between the site of inflammation and development of fibrosis is seen *in vivo* (Sorensen et al., 1984; Tsukamoto et al., 1990; Constandinou et al., 2005; Iredale, 2007). Liver inflammation activates hepatic stellate cells (HSCs) to a myofibroblastic phenotype, the main source of hepatic collagens in fibrosis (Friedman, 2008). Activated HSCs remodel the local ECM from its normal low density basement membrane-like consistency to one that is three- to fivefold more dense, with high collagen content (Friedman, 2000; Schuppan et al., 2001). With progressing fibrosis, ECM stiffness increases, thereby reducing liver elasticity (Wang et al., 2009). There is growing interest in the role of ECM stiffness not only as a consequence of fibrosis, but also as a contributor to fibrogenesis (Georges et al., 2007; Wells, 2008). Diagnostic technologies of liver stiffness, such as ultrasound or magnetic resonance elastography, are emerging, non-invasive alternatives to biopsy (Castera et al., 2005; Huwart et al., 2006; Takeda et al., 2006; Yin et al., 2007).

Despite *in vitro* evidence that matrix stiffness plays a determinant role in the phenotype of most adherent cells, there is little evidence that these findings apply *in vivo*. It is also unclear how macro-level tissue changes affect individual cells, which only mechanosense over short distances (Wells, 2008). Structural changes in cirrhosis can be extensive before the onset of functional hepatic decompensation; thus, it is also unclear how hepatocytic or ECM changes may play a role in shifting parenchymal cell function toward organ failure. Our liver fibrosis ABM (LFABM) is an *in silico* model that begins from simulation of healthy liver tissue, and, in response to toxic injury, progresses to a fibrotic phenotype characteristic of cirrhosis. In the LFABM, local cell mechanics and fibrotic development lead to overall matrix stiffness in the liver tissue.

Computational (*in silico*) modeling provides a means of addressing such questions, where *in vivo* and *in vitro* models are either inadequate or infeasible. In particular, translational systems biology aims to use computational methods to generate and test hypotheses regarding dynamic, complex disease processes (Vodovotz et al., 2008). One such method is the use of agent-based models (ABMs) to integrate local interactions to recapitulate overall dynamic changes in the referent biological system, thereby facilitating the generation of mechanistic hypotheses regarding emergent spatial or temporal patterns that often result in biological systems (An et al., 2009). ABMs have been used to gain clinical insights into several areas of clinical interest (An, 2004; Segovia-Juarez et al., 2004; Mi et al., 2007; Li et al., 2008; Brown et al., 2011). In the field of liver research, ABMs have been used to test hypotheses regarding the mechanistic details of hepatic drug disposition (Park et al., 2009), specific pathways for dioxin-induced toxicity (Bhattacharya et al., 2012), and dose–response across the sinusoidal network (Wambaugh and Shah, 2010). All of these model the liver from a pharmacological perspective. ABMs can also be used to explore coordinated cell behavior, such as during short-term liver regeneration (Hoehme et al., 2010). In our work, we present an ABM that simulates the development of chronic liver inflammation and fibrosis based on both molecular interactions and mechanical forces, by employing a multiscale modeling approach in which phenomenological rules are combined with biomechanical rules (Vodovotz et al., 2008; Meier-Schellersheim et al., 2009). Key predictions regarding emerging histological patterns were validated against experimental data from CCl_4_-treated rats (Grimm et al., 2005). In addition, two theoretical anti-fibrotic therapies were compared *in silico*.

## Materials and Methods

### Agent-based model

The LFABM consists of agents representing parenchymal cells (hepatocytes, live and dead), inflammatory cells (Kupffer cells), collagen-producing cells (HSCs and portal fibroblasts), and structural elements that define lobules (portal triads and septa). Agents can produce diffusible factors [tumor necrosis factor alpha (TNF-α), transforming growth factor beta 1 (TGF-β1), high mobility group box protein 1 (HMGB1)] that are subject to degradation, and can affect each other, or other agents. The properties of these agents and their interactions with each another are governed by rules obtained from the published literature. Details of these rules are available in Supplementary Material and a brief overview is provided in the Section “Modeling Cellular Interactions and Inflammation.”

All agents occupy space, and have the ability to identify other agents with which they come in contact. Agents representing hepatocytes, dead hepatocytes, septa, portal triads, and collagen are considered to contribute to tissue mechanics, and therefore possess the property of collision. Collision is defined in the context of our LFABM as the ability of an agent to occupy its own space, and exert force upon another agent when encountered. This property is important for biological fidelity to tissue mechanics. Since Kupffer cells, HSCs, portal fibroblasts, and myofibroblasts are not structural components of the organ tissue, these agents are assumed not to contribute to tissue mechanics by their physical presence, and therefore do not possess the property of collision. Consequently, these agents can co-exist in the same space, and our model would not capture any changes in size or elasticity due to physical pressure from inflammatory infiltrates.

The LFABM was implemented using Simple Platform for Agent-based Representation of Knowledge (SPARK) (Solovyev et al., 2010). Source code for the model is available for download at http://www.pitt.edu/~cirm/spark/models/LFABM.zip (Tutorials for SPARK and other ABM examples are freely available at http://www.pitt.edu/~cirm/spark).

### Animal model for ABM validation

In order to observe the histological pattern of fibrosis development over time, we obtained slides from an animal model of experimentally induced fibrosis. All procedures performed on animals were approved by the University of Pittsburgh Animal Care and Use Committees. Liver fibrosis was induced in male Lewis rats using CCl_4_ as described previously (Kobayashi et al., 2000; Liu et al., 2012).

Briefly, liver cirrhosis was induced beginning in 4-week-old inbred male Lewis rats, weighing 100–130 g, using Phenobarbital (Sigma Chem. Co., St. Louis, MO, USA) and carbon tetrachloride (CCl_4_, Sigma) (Kobayashi et al., 2000). Rats were given Phenobarbital (0.5 g/l) added to the drinking water. Starting 2 weeks later, CCl_4_ (diluted 1:9 in the olive oil) was administered by gavage on a full stomach twice a week. Following an initial dose of 0.2 ml/kg, each subsequent dose was adjusted weekly on the basis of changes in body weight. If the body weight increased or remained unchanged, CCl_4_ was continued at 0.2 ml/kg twice weekly. When body weight decreased by 1–5 g, the dose of CCl_4_ was reduced to 0.15 ml/kg, and if body weight decreased by 6–10 g the CCl_4_ was reduced to 0.1 ml/kg. In rats that lost more than 10 g of body weight, CCl_4_ was withheld until reassessment 1 week later. All animals receiving CCl_4_ were observed for 4 weeks after receiving their last dose of CCl_4_ to eliminate the acute effects of toxin exposure before any analysis was performed. Animals receiving CCl_4_ over 26–28 weeks generated cirrhosis that produced irreversible liver failure, and these animals died approximately 2–4 weeks after the 4-week observation period with progressive worsening of liver function. Animals with cirrhosis without liver failure received 13–14 weeks of CCl_4_. Laboratory tests and ascites resolved quickly in all of these animals after the 4 week observation period after discontinuing CCl_4_.

Masson’s trichrome stain was applied to distinguish collagen deposits.

### Modeling the liver lobules

**Figure 1:**
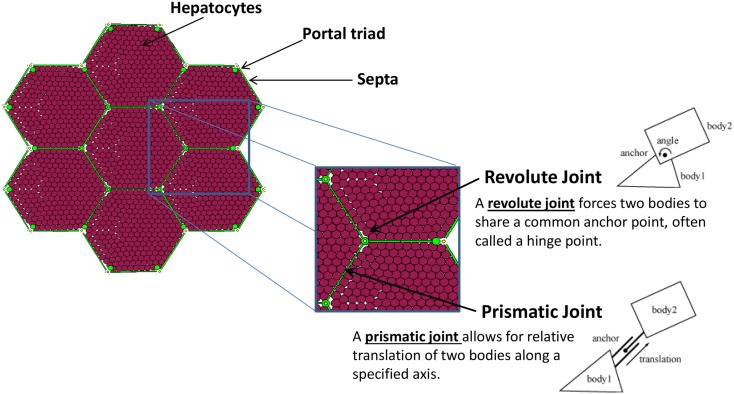
**The structural elements comprising the tissue framework**.

A patch of liver tissue was modeled as a two-dimensional monolayer of 3,857 hepatocytes (see Model Calibration and Parameter Estimation) arranged in lobules, as seen in Figure 1. Each septum, forming an edge of a lobule, was modeled by two boundary agents placed side by side, and connected with a prismatic joint. This type of joint allows the entities to slide along each other, if free to move. Each septum was connected to its adjacent portal triad by a revolute joint, which allows rotation around the portal triad, if free to move. Freedom of motion was determined by the relative physical forces exerted by tissue components.

### Modeling cellular interactions and inflammation

Hepatocytes are known to be long-lived under normal, healthy conditions, and have rapid regenerative capacity to replace hepatocytes lost due to resection or other means (Roskams, 2008). In the LFABM, agents representing hepatocytes have long lifespans and monitor their neighboring spaces. If empty space is detected, the hepatocyte replicates to fill that space. If, however, excess collagen is detected, the hepatocyte cannot replicate more than twice. This rule was incorporated to simulate suspected replicative senescence from observations in our previous work (Liu et al., 2012).

**Figure 2:**
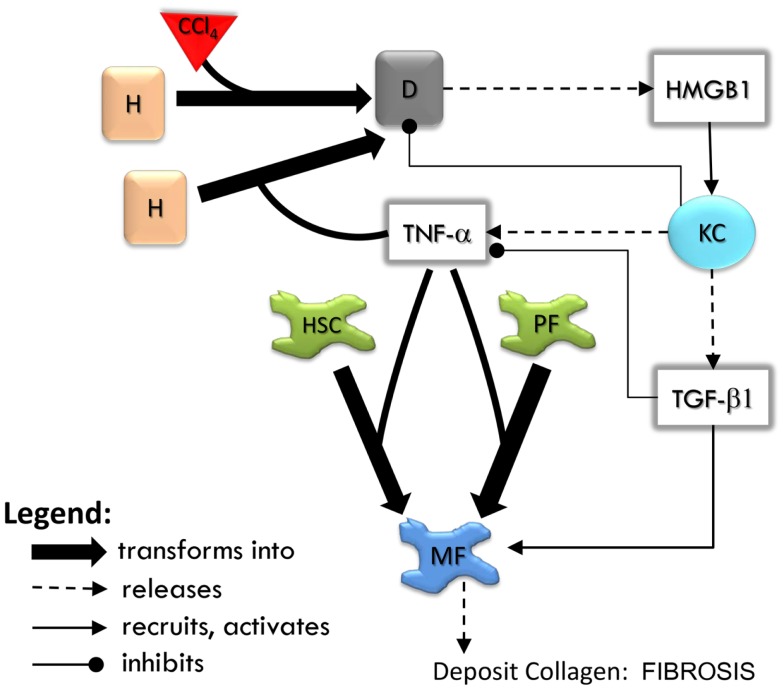
**An overview of agent interactions in the model**. H, hepatocyte; D, dead hepatocyte; KC, Kupffer cell; HSC, hepatic stellate cell; PF, portal fibroblast; MF, myofibroblast. Diffusible factors TNF-α, tumor necrosis factor alpha; TGF-β1, transforming growth factor beta 1; HMGB1, high mobility group box protein 1. Detailed rules are available in Supplementary Material.

The sequence of events in the LFABM is shown in Figure 2. Administration of CCl_4_ in animal models causes centrilobular necrosis of hepatocytes (Stachura et al., 1981). In our model, pulses of centrilobular toxicity transform hepatocytes into dead agents. Biologically, Kupffer cells survey the area and phagocytize dead cells and become activated to produce cytokines in the process (Edwards et al., 1993). In the LFABM, upon encountering a dead agent, Kupffer cells phagocytize and become activated to produce TNF-α (a canonical pro-inflammatory cytokine) and TGF-β1 (a canonical anti-inflammatory cytokine) (Martinez et al., 2008). Biologically, inadequate clearance of dead cells can lead to the release of damage-associated molecular pattern (DAMP) molecules such as HMGB1 (Scaffidi et al., 2002; Bell et al., 2006). DAMPs attract monocytes and neutrophils to the liver. Correspondingly, in the LFABM, inadequate clearance of dead agents leads to accumulation of HMGB1, with subsequent recruitment and transformation of monocytes to activated Kupffer cells. All parameters are set with probabilistic ranges.

High mobility group box protein 1 and TNF-α are potent stimulants of portal fibroblasts and HSCs, inducing their transformation to myofibroblasts (Knittel et al., 1997; Kao et al., 2008). TGF-β1 is known to induce myofibroblastic proliferation and deposition of ECM components (primarily type 1 collagen) by these cells (Maher and McGuire, 1990; Friedman, 2000). In the LFABM, agents representing portal fibroblasts and HSCs are activated and transformed to myofibroblast agents when they detect TNF-α. When they detect TGF-β1, myofibroblast agents proliferate and deposit collagen to existing ECM structure (Thannickal et al., 2003).

### Model calibration and parameter estimation

Hepatocytes account for approximately 60% of the total number of cells found in the liver, whereas Kupffer cells account for 15% and HSCs account for 5% (Malarkey et al., 2005). The initial number of hepatocytes in the LFABM was set by an automated process of filling hepatocytes per lobule in a spiral fashion, beginning at the center, and then allowing their collision and replication properties to fill up the lobule uniformly. For the simulation size presented in this paper, this number emerged to be 3,857. Considering this to represent 60% of the cell population of the liver, the number of Kupffer cells was initialized to 964, and the number of HSC to 321.

The agents were chosen to represent broad biological functions: the Kupffer cell was a representative inflammatory cell, TNF-α was a representative pro-inflammatory cytokine, and TGF-β1 was a representative anti-inflammatory/pro-fibrotic cytokine. Agent rules follow biological function, but not every intermediate is modeled. For example, exposure to above-threshold levels of TNF-α causes hepatocyte death in the LFABM. This bystander effect is biologically mediated by T-cell activation, which is downstream of Kupffer cell activation; however, we do not explicitly model T-cells, instead treating the effect of TNF-α on hepatocytes as a “lumped parameter” (Bhattacharya-Ghosh et al., 2012). Parameters for lifespans and proliferation rates were calibrated such that, in the absence of external perturbation, baseline equilibrium was maintained for each agent type in the system. All of the parameter values can be found in Supplementary Material.

### Simulated elastography measurement

A number of non-invasive techniques for evaluating liver fibrosis are currently in development. Some of these approaches use ultrasound or magnetic resonance elastography to quantify changes in the stiffness of liver tissue as a measure of fibrosis progression (Castera et al., 2005; Huwart et al., 2006; Takeda et al., 2006; Yin et al., 2007). In our simulations, a measure of tissue elasticity was obtained by calculating the average displacement of all internal nodes (each node that has three adjoining septa) one time step after a centrally (inward) directed impulse was applied to every internal node, with all outer nodes (nodes that are on the outer border of our simulated tissue patch) held immobile. A higher displacement value would indicate a more pliable condition of the tissue. Since this measurement requires an external perturbation that could affect later states of the simulation, a copy of the current state of the simulation was first saved, and then the impulse applied to this copy. In this case, “state” refers to the current positions of all agents, which contribute to tissue mechanics, as well as their relevant physical forces. In order to make up for the lack of compressibility – a natural biological quality of hepatocytes *in vivo* – of the cellular agents, a slight reduction in the diameter of each hepatocyte and dead agent was applied uniformly before applying the impulse. This allowed compression of the tissue even when fully packed with hepatocytes or dead agents. After the impulse was applied, and elastic displacement measured, this copy of the state was discarded.

### Statistical analysis

All simulation plots are presented as mean ± SD, using SigmaPlot 9.0 from Systat Software, Inc., San Jose, CA, USA (www.sigmaplot.com).

## Results

Of the various experimental models utilized in the study of liver fibrosis and cirrhosis, carbon tetrachloride (CCl_4_)-induced cirrhosis in rodent livers is considered to be closest to human cirrhosis in terms of morphology and pathophysiology (Perez Tamayo, 1983; Wu and Norton, 1996; Onori et al., 2000). Intoxication with CCl_4_ results in hepatocyte damage, necrosis, inflammation, and fibrosis, which spreads to link the vascular structures that feed into the hepatic sinusoids, leading to cirrhosis over 8–12 weeks (Constandinou et al., 2005). Accordingly, the *in silico* development of chronic inflammation and fibrosis in a patch of liver was verified against a histological time course obtained from the liver of rats exposed to CCl_4_.

**Figure 3:**
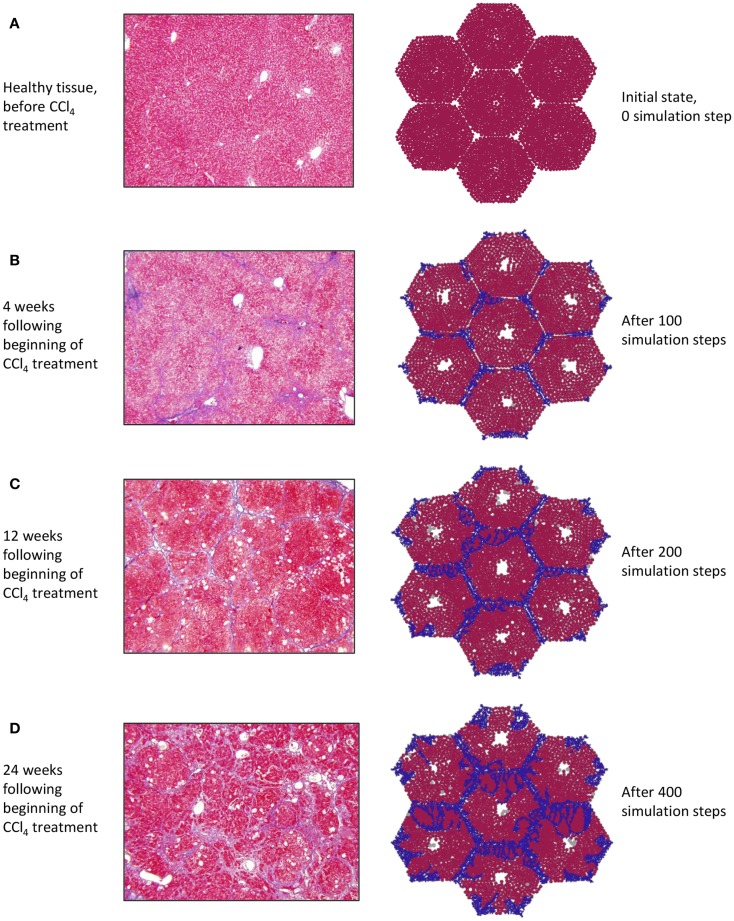
**(A–D)** Progression of fibrosis in experimentally obtained sections (left column) vs. in ABM simulation (right column); collagen appears blue in Masson’s trichrome stain (left), collagen agents in the model are also blue (right).

At baseline, both tissue sections stained with Masson’s trichrome (Figure 3A, left) and the simulation (Figure 3A, right) show a uniform texture across hepatocyte-filled lobules. As hepatocytes died in response to repeated CCL_4_ administration, persistent damage led to continuous inflammatory activation (Figure 3B), loss of hepatocytes, and the beginnings of collagen deposition – first in the periportal region, then extending across septa to form bridging fibrosis. With continued inflammation, collagen deposits grew and the lobules appeared more deformed, with some regenerative nodules forming as fibrous bands beginning to separate out smaller sections of parenchyma (Figures 3C,D).

**Figure 4:**
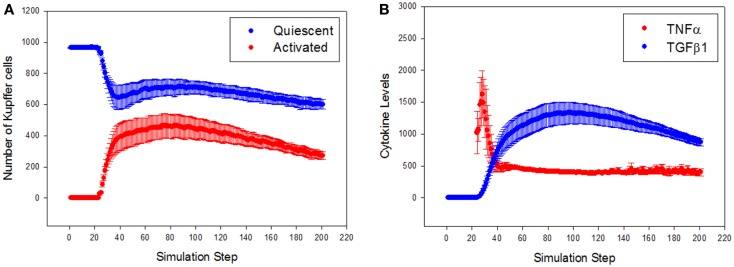
**General trajectory of Kupffer cell activation and cytokine production in simulations (*n* = 10, mean ± SD)**. **(A)** Resident Kupffer cells are at first quiescent (blue), but as inflammation progresses they are activated (red) and drive further recruitment of Kupffer cells; **(B)** the trajectory of TNF-α (red) and TGF-β1 (blue) as inflammation progresses. Activated Kupffer cells are at first dominated by an M1 phenotype, as demonstrated by the early peak of TNF-α, followed by a later, longer peak of TGF-β1 (transition to a domination of M2 phenotype).

To characterize the quantitative rather than qualitative behavior of the ABM, the predicted dynamics of cell populations and cytokine production were assessed. Resident Kupffer cells were activated by the CCL_4_-induced damage, and a concomitant rise in activated Kupffer cells and concurrent recruitment of further Kupffer cells were observed (Figure 4A). The activation of Kupffer cells slows down as the simulation progresses, but then stabilizes to a steady state after about 300 simulation time steps (Figure S1 in Supplementary Material). The Kupffer cell population was initially dominated by an M1 phenotype, characterized by release of TNF-α (Figure 4B). As Kupffer cells phagocytized dead hepatocytes, their phenotype shifted toward an M2 phenotype, characterized by release of TGF-β1.

**Figure 5:**
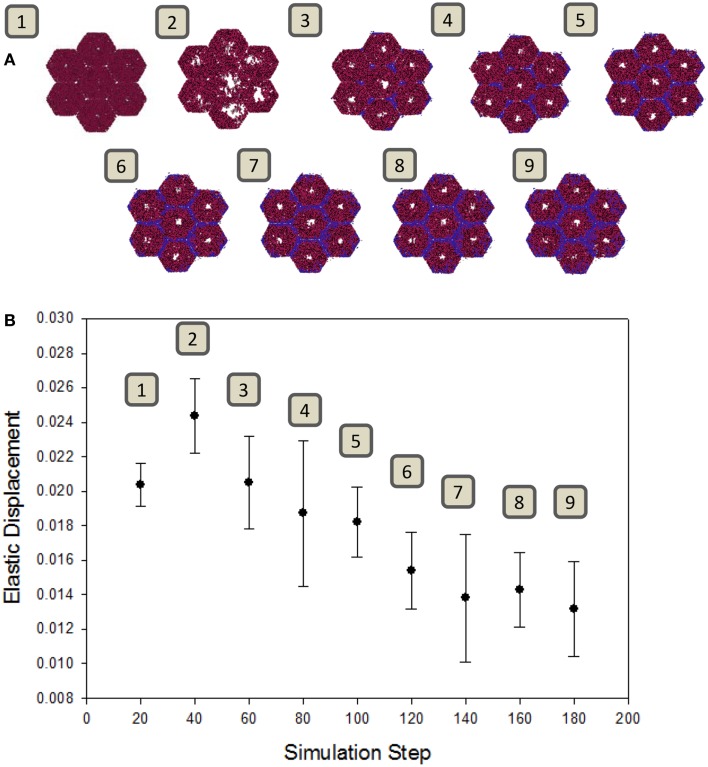
**Change in tissue elasticity with the progression of fibrosis**. **(A)** Snapshots of the model at intervals of 20 simulation time steps. **(B)** Elastic displacement measured at each of the same time points of simulations (*n* = 10, mean ± SD), as described in Section “Materials and Methods.” Units are relative to set simulation space.

A well-known consequence of fibrosis is an overall loss of the liver’s natural elasticity (Wang et al., 2009). To determine if the ABM exhibits this global behavior, a simulated elastography measurement was utilized. The observed change in elastic displacement, when measured periodically during a simulation, is shown in Figure 5. After an initial increase in tissue pliability, the liver steadily becomes stiffer over time, as fibrotic bands begin to appear. This behavior is consistent with clinical observations of fibrotic livers, where the mechanical properties of the liver tissue were found to be correlated with the extent of fibrosis (Yin et al., 2007).

**Figure 6:**
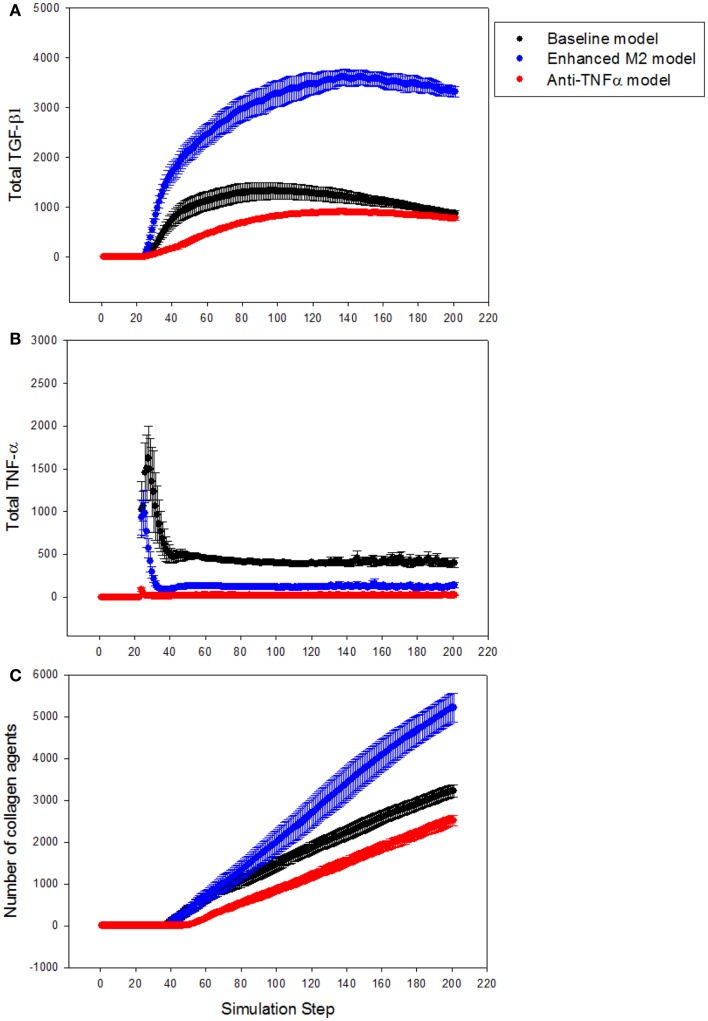
**Effect of anti-TNF treatment or M2 enhancement of Kupffer cells in the simulations (*n* = 10, mean ± SD)**. **(A)** TGF-β1 levels in the model with anti-TNF treatment (red), and in the model with enhanced M2 behavior (blue), compared to TGF-β1 levels in the baseline model (black); **(B)** TNF-α levels in the model with anti-TNF treatment (red), and in the model with enhanced M2 behavior (blue), compared to TNF-α levels in the baseline model (black); **(C)** growth of collagen in the anti-TNF-treated model (red), and in the model with enhanced M2 behavior (blue), compared to growth of collagen in the baseline model (black).

Next, the LFABM was used to test specific hypotheses regarding two potential anti-fibrotic therapies: modulation of M1/M2 Kupffer cell phenotype, and the administration of neutralizing anti-TNF-α antibodies. Although it is well-established that Kupffer cells play a key role in the pathogenesis of liver fibrosis, their participation has classically been associated with hepatic inflammation and activation of HSCs (Bataller and Brenner, 2005). Prior studies have explored the efficacy of anti-TNF-α treatments to reduce hepatic fibrosis, with some experimental evidence suggesting that inhibition of TNF-α signaling during liver injury may be efficacious (Bahcecioglu et al., 2008; Rockey, 2008). Kupffer cells that have differentiated to an M2 phenotype, characterized by release of the anti-inflammatory cytokine TGF-β1, also inhibit TNF-α signaling. However, experimental evidence has shown that M2 Kupffer cells can promote fibrogenesis (Lopez-Navarrete et al., 2011). To test the effect of these two mechanisms of anti-inflammatory treatment on the growth of fibrosis, the growth of collagen in the LFABM was examined under two new conditions: first, in response to the presence of an anti-TNF-α treatment (simulated by increasing the degradation rate of local TNF-α); second, in response to increased production of TGF-β1 by the Kupffer cell agents (thereby simulating enhanced M2 activation). TGF-β1 levels were reduced in anti-TNF-α simulations (Figure 6A, red), and elevated in the simulations of enhanced M2 behavior (Figure 6A, blue). TNF-α levels were reduced in both the anti-TNF simulations (Figure 6B, red) and the simulations of enhanced M2 behavior (Figure 6B, blue). The amount of collagen in the anti-TNF simulations (Figure 6C, red) was lower than untreated baseline, while the simulations of enhanced M2 behavior showed substantially greater accumulation of collagen (Figure 6C, blue) compared to untreated baseline.

## Discussion

This manuscript describes a multiscale ABM developed in order to simulate the development of chronic liver inflammation and fibrosis. This LFABM simulates key cellular and molecular processes of inflammation and fibrosis, as well as behavior (overall elasticity) at a tissue scale, ultimately generating predictions regarding dynamics of cell populations, patterns of tissue fibrosis, and lobular structure, as well as overall mechanical and structural changes (Meier-Schellersheim et al., 2009). The LFABM was verified through a qualitative pattern-oriented analysis, wherein emergent patterns are seen as defining characteristics of a system, and the ability of an ABM to recreate these patterns is indicative of its value in generating and testing hypotheses regarding system-level properties (Grimm et al., 2005). The development of fibrosis first periportally, then bridging across septa, and then eventually leading to regenerative nodules and lobular deformation was observed both in the LFABM and in a histological time course obtained from CCL_4_-treated cirrhotic rats. This pattern of collagen deposition emerged concurrent to the known biological time course of inflammation, consisting of an early pro-inflammatory peak, followed by a slower, late rise in anti-inflammatory/pro-fibrotic mediators. As fibrosis progressed, the simulated tissue overall became less pliable, analogous to the increase in tissue stiffness seen clinically as well as experimentally in CCl_4_-treated rats (Yin et al., 2007; Wang et al., 2009).

The primary motivation in building this LFABM was to leverage the power of simulation experiments on a biologically realistic system to examine the effects of potential anti-fibrotic strategies. Thus, the model was used to test hypotheses *in silico* regarding the role of Kupffer cells and TNF-α in the progression of fibrosis in the liver. There is controversy over whether Kupffer cells are primarily involved in cirrhosis as promoters of inflammation or fibrogenesis. An experimental approach to address this question selectively stimulated alternate activation in mouse Kupffer cells, and found that collagen levels were higher in these mice when treated with CCl_4_ despite markedly lower levels of inflammatory cell populations in these animals (Lopez-Navarrete et al., 2011). Others have observed that anti-inflammatory treatment could attenuate necro-inflammation, and thereby fibrosis, in the CCl_4_-treated liver. One such study involved the use of infliximab, an anti-TNF-α agent, and reported lower fibrosis scores due to this treatment (Bahcecioglu et al., 2008). Our results are consistent with these latter observations. In the study using infliximab, the authors reported no significant difference in serum TNF-α levels, while observing relatively reduced levels of TGF-β1. It is possible, therefore, that experimental suppression of TNF-α affects fibrosis through mediation of TGF-β1 levels; similar effects were suggested in an earlier ABM of inflammation in the setting of chronic, non-healing diabetic foot ulcers (Mi et al., 2007).

A limitation of the multiscale modeling approach described herein involves the abstraction across different scales of biological organization. This abstraction includes choosing representative cells and cytokines to represent overall mechanism, and using “lumped parameters” to summarize the main effects of biological processes (Bhattacharya-Ghosh et al., 2012). This abstraction means that, inevitably, some mechanistic details of the system being modeled are sacrificed. However, such simplification allows us to define the main functional modules that lead to multiscale emergent behaviors at the tissue level, and the liver as a whole. In a similar vein, prior ABMs of diabetic foot ulcers (Mi et al., 2007) and particulate-injured lung (Brown et al., 2011) abstracted inflammatory cells as well as pro- and anti-inflammatory cytokines in a manner similar to that depicted herein. In addition, the LFABM was not calibrated against any specific time course of liver injury, though simulations did match the general progression of CCl_4_-induced histological changes. Future versions of this model could calibrate the time course of simulated inflammation to clinically observed time courses, with the goal of suggesting therapies for fibrosis in a disease-specific, and perhaps also patient-specific manner.

## Conflict of Interest Statement

The authors declare that the research was conducted in the absence of any commercial or financial relationships that could be construed as a potential conflict of interest.

## Supplementary Material

The Supplementary Material for this article can be found online at http://www.frontiersin.org/Journal/10.3389/fbioe.2014.00018/abstract

## Abbreviations

ABM, agent-based model; CCl_4_, carbon tetrachloride; DAMP, damage-associated molecular pattern; ECM, extracellular matrix; HMGB1, high mobility group box protein 1; HSC, hepatic stellate cell; LFABM, liver fibrosis agent-based model; TGF-β1, transforming growth factor beta 1; TNF-α, tumor necrosis factor alpha.
