# Supplementary material for: A Multiscale Agent-Based in silico Model of Liver Fibrosis Progression
Source: Front Bioeng Biotechnol. 2014 May 30;2:18. doi: 10.3389/fbioe.2014.00018 (PMC4126446; doi:10.3389/fbioe.2014.00018)
Supplement: Supplementary file 1 [file Data_Sheet1.DOCX]

**SUPPLEMENTARY MATERIALS**

**ABM Rules**

**AGENTS**

**Kupffer cell.** (Called *Macrophage* in the model files.)

1. [Initialization] Can be active or inactive. All newly created Kupffer cells are inactive and have the lifespan of 0-200 ticks. The radius is 0.04 units.
2. [Active or inactive] Randomly moves with the speed of 0.1 units per tick.
3. [Active or inactive] Phagocytizes dead hepatocyte cells.
4. [Inactive] Activated if the value of HMGB1 is greater than 0.1. The lifespan of an activated cell is increased by 50 ticks.
5. [Active] Produces TNF and TGF. New values are computed with the equations:
   TNF(new) = TNF(old) + 1 / (TGF(old) + 0.01),
   TGF(new) = TGF(old) + 0.2 * (the number of phagocytized cells).

**Dead hepatocyte cell.** (Called *Dead* in the model files.)

1. Produces HMGB1 every tick with the equation
   HMGB1(new) = HMGB1(old) + 0.2.

**Hepatocyte cell.** (Called *Hepatocyte* in the model files.)

1. [Initialization] The initial lifespan is 100-600 ticks. The radius is 0.08 units.
2. Dies (becomes a dead cell) if the value of CCL4 is higher than 0.1.
3. [With 50% probability] If the value of TNF is greater than 4, a hypatocyte cell dies and adds 0.1 to the current value of HMGB1.
4. Randomly chooses a space around and checks if it is empty. When an empty space is detected, a hepatocyte cell tries to replicate to fill that space. A hepatocyte cell can replicate many times (up to 100 times) when there is no collagen nearby. It can replicate only 2 times in the presence of collagen.

**Central vein.** (Called *CentralVein* in the model files.)

1. [Initialization] The radius is 0.05 units.
2. Every 10 ticks adds 1 to the current value of CCL4.

**Portal agent.** (Called *Portal* in the model files.)

1. [Initialization] The radius is 0.1 units.
2. [With 20% probability] Creates a Kupffer cell.
3. [With 20% probability] If the total global value of HMGB1 is greater than 10, creates a Kupffer cell.

**Portal fibroblast.** (Called *PortalFibroblast* in the model files.)

1. [Initialization] The initial lifespan is 50-100 ticks. Can be active or inactive. All newly created portal fibroblasts are active.
2. [Active] If the value of TGF is greater than 0.3, a portal fibroblast deposits collagen and becomes inactive.

**Myofibroblast.** (Called *Myofibroblast* in the model files.)

1. [Initialization] The initial lifespan is 0-40 ticks. The radius is 0.04 units.
2. Randomly moves with the speed of 0.1 units per tick.
3. If the value of TGF is greater than 0.3 and there are less than 200 myofibroblasts around, a myofibroblast proliferates with the probability (2 * (the TGF value))%.
4. If the value of TGF is greater than 0.3, a myofibroblast deposits collagen to existing ECM structures (it invokes the method *proliferate* for all collagen agents in the neighborhood). Every time new collagen is deposited, the lifespan of the myofibroblast is reduced by 10 ticks.

**Hepatic stellate cell (HSC).** (Called *StellateCell* in the model files.)

1. [Initialization] The initial lifespan is 0-50 ticks. The radius is 0.04 units.
2. Randomly moves with the speed of 0.1 units per tick.
3. If the value of TNF is greater than 0.3 or the value of HMGB1 is greater than 0.004, then a myofibroblast is created and the stellate cell dies.
4. Each tick, 3-13 stellate cells are created at random positions inside the lobules.

**Collagen.** (Called *Collagen* in the model files.)

1. [Initialization] The shape is a 0.05 x 0.03 rectangle.
2. Collagen is created by portal fibroblasts and myofibroblasts.
3. Collagen created by portal fibroblasts is attached to them.
4. Myofibroblasts create collagen which is attached to existing collagen.

**Boundary.** (Called *Boundary* in the model files.)

1. A structural agent which models edges of lobules. A boundary agent has a thin rectangular shape. Each septum, forming an edge of a lobule, is modeled by two boundary agents connected with a prismatic joint.

**Mediators**

**TNF**

1. Produced by activated Kupffer cells:
   TNF(new) = TNF(old) + 1 / (TGF(old) + 0.01).
2. Kills hepatocyte cells if the value of TNF is greater than 4.
3. Transforms a stellate cell into a myofibroblast if the value of TNF is greater than 0.3.
4. TNF diffuses in the following sense: every tick each data grid cell which models TNF shares 100% of its value with neighbors.
5. TNF evaporates in the following sense: every tick each data grid cell which models TNF is multiplied by 0.5.

**TGF**

1. Produced by activated Kupffer cells:
   TGF(new) = TGF(old) + 0.2 * (the number of cells phagocytized by the Kupffer cell).
2. Stimulates deposition of collagen by portal fibroblasts If the value of TGF is greater than 0.3.
3. Stimulates proliferation and deposition of collagen by myofibroblasts if the value of TGF is greater than 0.3.
4. TGF diffuses in the following sense: every tick each data grid cell which models TGF shares 100% of its value with neighbors.
5. TGF evaporates in the following sense: every tick each data grid cell which models TGF is multiplied by 0.8.

**HMGB1**

1. Produced by dead hepatocyte cells (they add 0.2 to the value of HMGB1 each tick).
2. Activates Kupffer cells if the value of HMGB1 is greater than 0.1.
3. Stimulates creation of Kupffer cells by portal agents If the total global value of HMGB1 is greater than 10.
4. Transforms a stellate cell into a myofibroblast if the value of HMGB1 is greater than 0.004.
5. HMGB1 diffuses in the following sense: every tick each data grid cell which models HMGB1 shares 100% of its value with neighbors.
6. HMGB1 evaporates in the following sense: every tick each data grid cell which models HMGB1 is multiplied by 0.5.

**CCL4**

1. Produced by central vein agents (they add 1 to the value of CCL4 every 10 ticks).
2. Kills hepatocyte cells if the value of CCL4 is higher than 0.1.
3. CCL4 diffuses in the following sense: every tick each data grid cell which models CCL4 shares 50% of its value with neighbors.
4. CCL4 evaporates in the following sense: every tick each data grid cell which models CCL4 is multiplied by 0.97.

**WORLD**

The world is a 12 units x 12 units square.

**Initialization**

1. The liver lobules are created. They are modeled with boundary and portal agents. Figure 1 shows the initial configuration.
2. Central veins are created at the centers of the lobules.
3. Hepatocyte cells are created in each lobule. The total initial number of hepatocytes is 3857.
4. Kupffer cells are created at random positions inside the lobules. The total initial number of Kupffer cells is 964.
5. Stellate cells are created at random positions inside the lobules. The total initial number of stellate cells is 321.
6. Portal fibroblasts are created inside portal agents. 6 portal fibroblasts are created for each portal agent.


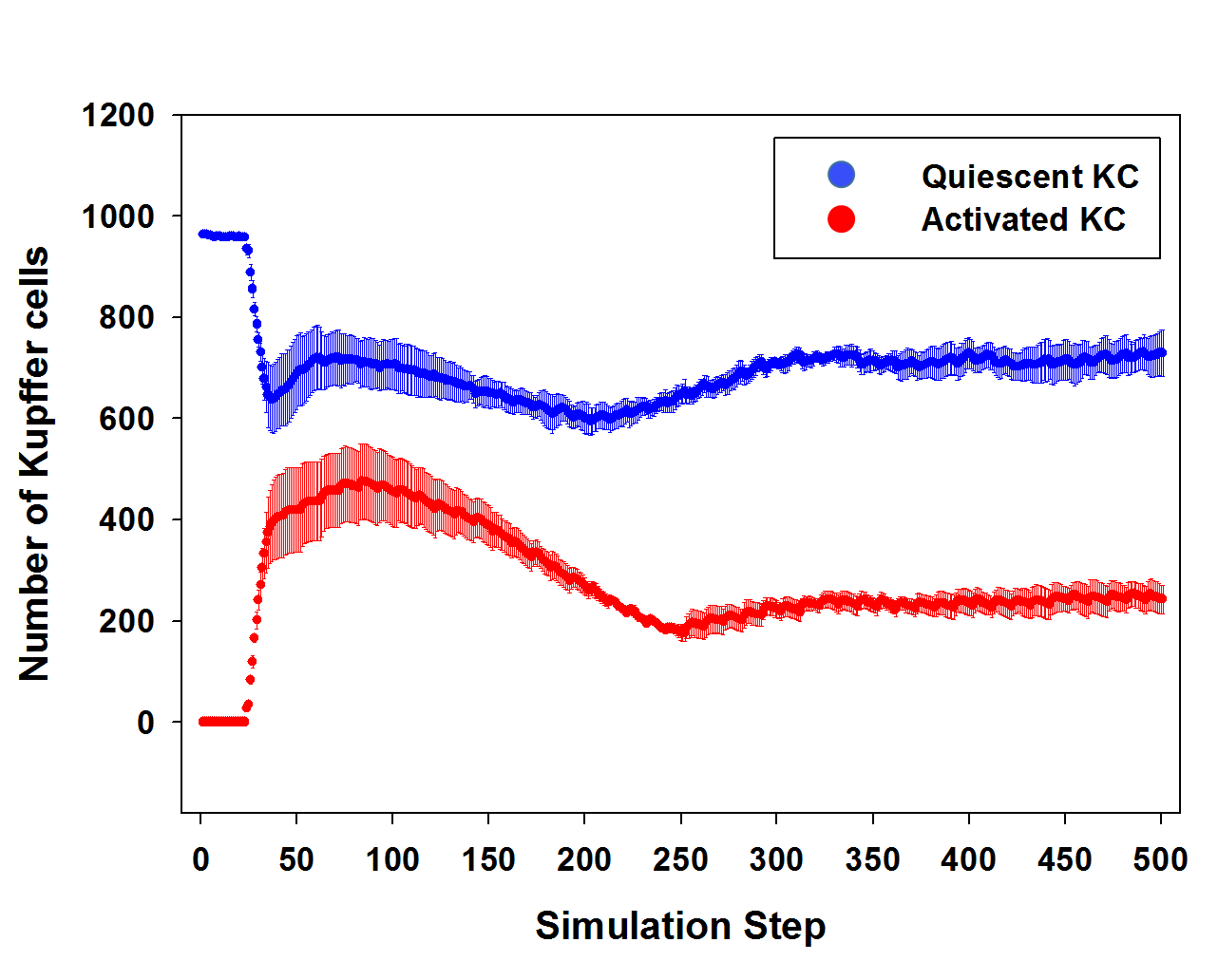


**Supplementary Figure 1**: Kupffer cell agents in the liver ABM as simulation progresses, upto 500 simulation time steps (n = 5, mean ± standard deviation). The number of agents reach steady state in later time points.
